# Analysis of traditional feeding practices and stunting among children aged 6 to 59 months in Karanganyar District, Central Java Province, Indonesia

**DOI:** 10.1186/s12887-023-04486-0

**Published:** 2024-01-08

**Authors:** Yuly Astuti, Seung Chun Paek, Natthani Meemon, Thammarat Marohabutr

**Affiliations:** Department of Society and Health, Faculty of Social Sciences and Humanities, Mahidol University, 999 Phutthamonthon 4 Road Salaya, Nakhon Pathom, 73170 Thailand

**Keywords:** Stunting, Traditional behavior, Feeding practices, Cultural beliefs, Primary data, Indonesia

## Abstract

**Background:**

Traditional feeding practices are widespread in Indonesia. Therefore, using traditional feeding practices commonly used among mothers, this study examined the association between these practices and stunting along with other relevant factors (i.e., sociodemographic factors, feeding practices, vaccination status, and place of residence).

**Methods:**

This cross-sectional study was conducted in Karanganyar District, Central Java Province. Data from a total of 706 children aged 6 to 59 months (352 children with stunting and 354 children without stunting) were obtained from the medical records of 10 primary health care units (PHCUs) in 9 subdistricts. Descriptive analysis and binary logistic regression (BLR) were performed to explore the association between the dependent (stunting) and independent variables.

**Results:**

The BLR results from children 6 to 59 months indicated that children of mothers with food restrictions during pregnancy (AOR = 5.87, 95% CI: 3.03, 11.38), children with prelacteal feeding (AOR = 4.27, 95% CI: 2.16, 8.41) and children with food restrictions (AOR = 7.74, 95% CI: 1.22, 49.16) were more likely to experience stunting. Those from children 6 to 23 months revealed that food restrictions during pregnancy (AOR = 17.55, 95% CI: 2.86, 107.80) and prelacteal feeding (AOR = 10.58, 95% CI: 2.06, 54.41) were related to stunting. The reasons for traditional feeding practices were related to cultural beliefs. For example, mothers believed that red meat could cause high blood pressure; thus, the consumption of red meat could trigger miscarriage or bleeding during delivery. In addition, this study showed that low sociodemographic status, inappropriate feeding practices, incomplete vaccination, and residence in rural areas were related to stunting.

**Conclusions:**

The findings reflect the importance of education for mothers to correct misconceptions of traditional feeding practices. The government should strengthen counseling services in PHCUs to improve mothers’ knowledge of and attitudes toward appropriate feeding practices. Additionally, public relations practices through the mass media should continue for family members, especially senior members, as they influence mothers’ autonomy in decision-making regarding feeding practices in Indonesia.

## Background

Stunting is a major public health issue in the Southeast Asia region [1]. According to a joint report by UNICEF, WHO, and the World Bank in 2020, approximately 24.1% of children aged under 5 years in the Southeast Asia region experienced stunting [2]. Stunting is primarily caused by chronic undernutrition and infection during pregnancy through the second year of life and continues until the age of 5 years [3, 4]. The short-term negative consequences of stunting include a low immune system, diminished physical development, and impaired cognitive function in children aged under 5 years [5, 6]. The long-term negative consequences of stunting include decreased school performance in childhood and an increased risk of chronic diseases in adulthood [6, 7]. In addition, stunting can be genetic in women. This means that women who experienced stunting can have stunted offspring [8, 9].

Among Southeast Asian countries, Indonesia has a persistently higher prevalence of stunting. In 2020, approximately 31.8% of children aged under 5 years were stunted, which was the second highest rate in the region after East Timor, with a rate of approximately 48.8% [2]. The government has tried to reduce the prevalence of stunting by including it in the national public health agenda since 2017 [10]; however, the reduction has been relatively slower than the government’s expectation [11]. From 2012 to 2020, the reduction in Indonesia was approximately 2.7 percentage points, from 34.5% to 31.8%. This decline was lower than that in Southeast Asia, which was approximately 3.6 percentage points, and far lower than that of 5 target countries (Lao PDR, Cambodia, Philippines, Myanmar, and Vietnam), which was approximately 5.8 percentage points for the same time period [2].

Factors related to stunting have been examined in numerous previous studies [4, 12–23]. Various socioeconomic conditions were shown to be associated with stunting, including household income level, maternal education and health status, and child birth weight, age and sex [4, 12, 13, 22, 23]. Optimal feeding practices, including exclusive breastfeeding, early initiation of breastfeeding within one hour after birth, and timely introduction of complementary foods, could lower the risk of stunting [14–17]. Furthermore, infections, including recurrent child infections, and vaccination, as well as environmental factors, including access to safe drinking water and sanitary conditions, were found to be determinants of stunting in children [18–21].

In Indonesia, traditional feeding practices are widespread among mothers due to culture and religion. According to field experts, certain practices are inappropriate for children and lead to stunting. For instance, some mothers perform prelacteal feeding by applying honey or sugar on their babies’ lips, as they believe that applying something sweet will bring good fortune to the babies in the future. Some mothers are also known to give rice water to their babies because they believe that its white color and thick texture have benefits similar to those of breast milk. In addition, an incomplete vaccination status, which puts children at higher risk of stunting, seems to be related to certain religious beliefs, since some Muslim mothers are doubtful of the impermissible ingredients (i.e., porcine-derived products) used in vaccines. Traditional feeding practices in this study refer to belief-driven feeding practices.

To the best of our knowledge, traditional feeding practices and incomplete vaccination have been relatively understudied in Indonesia because most previous studies conducted in Indonesia used secondary nationwide survey data sources [24–26]. Therefore, this study aimed to add to the body of knowledge on factors related to stunting among children aged 6 to 59 months. Specifically, along with the general factors evaluated in previous studies, we analyzed whether traditional feeding practices commonly used among Indonesian mothers are related to stunting and explored the reasons for these practices. Additionally, reasons for incomplete vaccination were explored.

## Methods

### Study design and area

This study employed a cross-sectional design with primary data. The data for this study were collected in Karanganyar District, Central Java Province, Indonesia, between May and September 2021. In 2021, Karanganyar District was categorized as one of the 100 high-risk districts for stunting by the Indonesian government [27]. Moreover, Karanganyar District has a relatively high stunting prevalence among the 100 high-risk districts. In 2018, the prevalence of stunting in the district was 28%, which was higher than the average stunting prevalence of 25.7% among the 100 districts [28, 29].

The district is approximately 565 km (km) from Jakarta, the capital of Indonesia. Administratively, it includes 17 subdistricts with a total of 21 primary health care units (PHCUs) [30].

The population of this study included all children aged 6 to 59 months who had registered medical records in all 21 PHCUs in the 17 subdistricts. The sample population included all children aged 6 to 59 months in 10 PHCUs in 9 subdistricts. In fact, we initially planned to recruit the study sample from all 21 PHCUs. However, during the data collection period, 11 PHCUs in 8 subdistricts were designated as restricted areas due to the COVID-19 pandemic, and accordingly, the study sample was recruited from the other 10 PHCUs in 9 subdistricts. The 9 subdistricts included 1) Karanganyar, 2) Tasikmadu, 3) Kebakkramat, 4) Gondangrejo, 5) Jaten, 6) Jatipuro, 7) Matesih, 8) Karangpandan, and 9) Mojogedang.

### Sampling technique

**Fig. 1:**
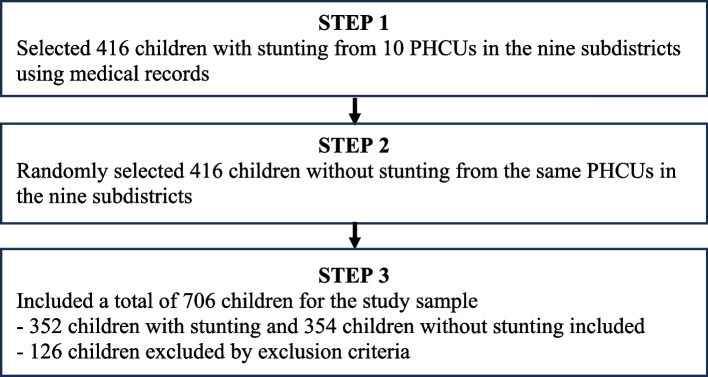
Flowchart of study sample selection

A total of 706 children aged 6 to 59 months were included in the study sample for analysis through the following three steps (Fig. 1):

Step 1. We visited all 10 PHCUs in the nine subdistricts and reviewed the latest medical records to identify children aged 6 to 59 months with stunting. Data on stunting status were obtained from medical records in April 2021 and we started data collection in May 2021. These children were determined using the WHO standard. A child height-for-age less than minus two standard deviations from the median of the WHO child growth standard was defined as stunting [31]. In this step, a total of 416 children with stunting were obtained. We cross-checked the maternal and child health handbook during actual data collection to verify the stunting status obtained from medical records.

These PHCUs used an anthropometry kit provided by the Ministry of Health, which includes a portable stadiometer to measure the height of children aged 1 to 5 years (Kenko®), an infantometer board to measure the height of children under 1 year of age (Metrisis®), a body measuring tape to measure the upper arm and head circumference of children aged 0 to 5 years (Seca®), a digital baby weight scale to measure the weight of children under 1 year of age (Sella®), and a digital weight scale to measure the weight of children aged 1 to 5 years (Camry®).

Step 2. We selected the same number of children aged 6 to 59 months without stunting from the medical records by using a simple random sampling method. To achieve this, we assigned numbers to the medical records of all children without stunting and selected them by the random number generated. In this step, a total of 832 children (416 children with stunting and 416 children without stunting) were obtained. The sampling method was applied by matching the ratio of children without stunting in urban and rural areas to that of children with stunting. Specifically, among the 416 children with stunting obtained in Step 1, 312 children (75%) and 104 children (25%) lived in rural and urban areas, respectively. However, we encountered a challenge due to the insufficient number of children with stunting in urban areas. As a result, we were unable to match the ratio of children without stunting in urban and rural areas to that of children with stunting.

Step 3. We visited all 832 children. Three exclusion criteria were additionally applied during the visits. First, children who lived in the subdistricts for less than 6 months (10 children without stunting) were excluded. Second, those with birth defects (4 children with stunting including 1 child with cleft lip/palate, 1 child with Down syndrome, and 2 children with congenital heart defects) were excluded. Third, the youngest child was selected if the mothers had more than one child (48 children with stunting and 5 children without stunting). In addition, during the visit, the mothers of 12 children with stunting and 47 children without stunting were excluded because they were found to be COVID-19-positive. After all steps, a total of 706 children (352 children with stunting and 354 children without stunting) were included.

### Data collection tools

Data were collected using a structured questionnaire through a face-to-face interview with the mothers of the 706 children. The questionnaire was developed by referring to the standard questionnaire developed by the Indonesian government and previous studies. Specifically, the Indonesian government standard questionnaire was used to develop questions, particularly on sociodemographic factors [32], community factors [32], early initiation of breastfeeding [33], exclusive breastfeeding [33], duration of breastfeeding [33], and immunization status [33]. And, previous studies were referred to develop questionnaires on other independent variables such as minimum dietary diversity [34], feeding frequency [34], and prelacteal feeding [16, 24]. In addition, questions related to traditional feeding practices, the reasons for the practices, and the reasons for incomplete vaccination were developed in consultation with field experts, including 5 midwives and 5 nurses in the PHCUs and 10 community health workers.

We and two trained research assistants conducted interviews in Bahasa, the national language of Indonesia, and it took approximately 45–50 min to complete the questionnaire. Training was provided to the two research assistants for two days prior to actual data collection and focused on how to approach the mothers and collect data. The research assistants were involved throughout the data collection process, from the pretest questionnaire to data collection.

In addition, data collection was carried out during the COVID-19 pandemic. We adhered to the government’s protocols for the prevention of COVID-19 as follows: (1) before conducting the survey, the researcher and research assistants reported their complete COVID-19 vaccination status to the District Health Office and the 10 PHCUs in the nine subdistricts; (2) the wearing of masks was mandatory during all interviews; (3) the COVID-19 rapid test was carried out for the researcher and research assistants, and the results were reported to the PHCUs on a daily basis; and (4) to comply with physical distancing regulations, only one researcher/research assistant and one community health worker remained together with the mother at one location during the interview.

### Questionnaire pretest procedure

Before actual data collection, a pretest questionnaire was administered. Specifically, we visited 1 PHCU in Sukoharjo District, the neighboring district of the study area, and randomly selected 36 children (18 children with stunting and 18 children without stunting) based on medical records, which accounted for approximately 5% of the total study sample. Then, we visited all 36 children and conducted face-to-face interviews with their mothers.

After the pretest, we adjusted the questions, particularly those related to food restrictions during pregnancy. For example, during the interview, some mothers responded that eating beef or mutton during pregnancy caused hypertension, miscarriage, and birth complications, which were not considered when the questionnaire was developed with the field experts. Accordingly, we included these unconsidered responses in the questionnaire. Additionally, some words in the prelacteal feeding questions, such as rice water, were changed from Bahasa to the local language for easier understanding by the mothers.

### Study variables

Stunting status, the dependent variable in this study, was measured as a binary variable: with and without stunting. As we mentioned previously in step 1 of the sampling technique, stunting status was secondary data obtained from medical records in 10 PHCUs. During actual data collection, we verified the stunting status by cross-checking with the maternal and child health handbook.

Independent variables were classified into four groups that included 1) sociodemographic factors, 2) feeding practices, 3) vaccination status, and 4) community factors. All independent variables were measured as categorical variables.

Sociodemographic factors included child age (6–11 months, 12–23 months, 24–35 months, and 36–59 months), child sex (male and female), child size (low birth weight [LBW] and normal birth weight), preceding birth interval (< 24 months, ≥ 24 months, and no previous birth), maternal education level (low = junior high school or below, middle = high school, and high = college or above), maternal working status (yes and no), and household income level (low =  ≤ 2 million Indonesian Rupiah [IDR], middle =  > 2 million– < 3.5 million IDR, and high =  ≥ 3.5 million IDR). The exchange rate of IDR to USD was 14,500 IDR/USD during the data collection period. The household income threshold for the low, middle, and high categories was approximately ≤ 137.9 USD, > 137.9– < 241.4 USD, and ≥ 241.4 USD, respectively. The categories of household income levels were determined based on the income distribution of the study sample.

Feeding practices included breastfeeding and complementary feeding practices. Breastfeeding practices included early initiation of breastfeeding (within 1 h after birth and after 1 h after birth), exclusive breastfeeding (yes and no), and duration of breastfeeding (current breastfeeding, < 23 months, and up to 23 months). Complementary feeding practices included minimum dietary diversity (< 4 food groups and ≥ 4 food groups) and feeding frequency (inadequate and adequate feeding frequency).

Additionally, traditional feeding practices included food restrictions during pregnancy (yes and no), food restrictions during breastfeeding (yes and no), food restrictions for children (yes and no), and prelacteal feeding (yes and no). Regarding food restriction, if mothers and children had food restriction for seafood, meat, and dairy products, they were classified into the yes group. For prelacteal feeding, mothers who gave their newborns honey, coffee, date syrup, rice water, and drinking water were classified into the yes group.

Vaccination status included basic vaccination status (complete and incomplete). According to the Indonesian Ministry of Health, complete vaccination requires 11 vaccines (one dose of hepatitis B [HB] vaccine, one dose of Bacille Calmette-Guerin (BCG) vaccine, three doses of Diphtheria-Tetanus-Pertussis [DTP] vaccine, Haemophilus Influenzae Type B [Hib] and HB vaccine, four doses of polio vaccine, one dose of inactivated poliovirus vaccine [IPV] vaccine, and one dose of measles-rubella [MR] vaccine) for children aged 0–9 months. Last, the community factor included the place of residence (rural and urban) [35].

Among these independent variables, birth weight, exclusive breastfeeding, and vaccination status were secondary data obtained from the maternal and child health handbook. This secondary information was then cross-checked with the mothers during the actual interview to verify its accuracy.

### Data analysis

A descriptive statistical analysis was conducted to provide summary statistics of the study sample and variables. In the analysis, a chi-squared test was performed to examine the bivariate association between each independent variable and stunting status. In addition, because the dependent variable was binary, a binary logistic regression (BLR) analysis was performed to measure the relationship between dependent (stunting) and independent variables. The odds ratio and its 95% confidence interval were used to determine the directional association and its statistical significance, respectively. Additionally, the Hosmer‒Lemeshow goodness-of-fit test was used to evaluate the performance of the BLR model. All statistical analyses in this study were conducted using IBM SPSS Statistics 26.

## Results

### Descriptive statistical analysis

Table 1 presents the results of the descriptive statistical analysis. Regarding sociodemographic factors, among the total sample of 706 children, 44.4% were aged 36–59 months. In addition, 25.1%, 22.1%, and 8.4% of the children were aged 24–35, 12–23, and 6–11 months, respectively. The study sample consisted of 52% males and 48% females. Approximately 13% of the children had LBW. Out of the total study sample, 62.7% had a birth interval ≥ 24 months, 31.1% had no previous birth, and 6.2% had a birth interval < 24 months. Regarding maternal education level, approximately 42% and 27% of the mothers had completed high school and college or above, respectively, and 30% had completed junior high school or below. Approximately 48% of the mothers were working. Approximately 40% of the children lived in a low-income household, and 30.2% and 29.3% lived in middle- and high-income households, respectively.

**Table 1:** Descriptive statistical analysis of children aged 6 to 59 months with and without stunting

Variables	Overall		Stunting status				*p* value
			**Children with stunting**		**Children without stunting**		
	**n**	**%**	**n**	**%**	**n**	**%**	
Sociodemographic factors							
**Child age**							< 0.001*
6–11 months	59	8.4	20	33.9	39	66.1	
Mean (SD)	70.09 (3.37)		66.50 (1.67)		71.94 (2.38)		
12–23 months	156	22.1	62	39.7	94	60.3	
Mean (SD)	78.23 (4.30)		75.26 (3.44)		80.17 (3.65)		
24–35 months	177	25.1	102	57.6	75	42.4	
Mean (SD)	86.42 (4.47)		83.50 (2.35)		90.39 (3.51)		
36–59 months	314	44.4	168	53.5	146	46.5	
Mean (SD)	97.93 (7.04)		93.40 (3.86)		103.13 (6.23)		
**Child sex**							0.010*
Male	367	52.0	200	54.5	167	45.5	
Female	339	48.0	152	44.8	187	55.2	
**Child size**							< 0.001*
LBW (< 2.5 kg)	90	12.7	86	95.6	4	4.4	
Normal birth weight (≥ 2.5 kg)	616	87.3	266	43.2	350	56.8	
**Preceding birth interval**							< 0.001*
< 24 months	44	6.2	40	90.9	4	9.1	
≥ 24 months	443	62.7	231	52.1	212	47.9	
No previous birth	219	31.1	81	37.0	138	63.0	
**Maternal education level**							< 0.001*
Low (junior high school or below)	212	30.0	179	84.4	33	15.6	
Middle (high school)	299	42.4	136	45.5	163	54.5	
High (college or above)	195	27.6	37	19.0	158	81.0	
**Maternal working status**							0.042*
Yes	342	48.4	184	53.8	158	46.2	
No	364	51.6	168	46.2	196	53.8	
**Household income level**							< 0.001*
Low (≤ 2 million IDR)	286	40.5	236	82.5	50	17.5	
Middle (> 2 million– < 3.5 million IDR)	213	30.2	78	36.6	135	63.4	
High (≥ 3.5 million IDR)	207	29.3	38	18.4	169	81.6	
Feeding practices							
**Early initiation of breastfeeding**							< 0.001*
Within 1 h	433	61.3	183	42.3	250	57.7	
After 1 h	273	38.7	169	61.9	104	38.1	
**Early initiation of breastfeeding**^**a**^							
Within 1 h	120	17.0	43	35.8	77	64.2	< 0.001*
After 1 h	95	13.5	59	62.1	36	37.9	
Children ≥ 24 months	491	69.5	250	50.9	241	49.1	
**Exclusive breastfeeding**							< 0.001*
No	410	58.1	300	73.2	110	26.8	
Yes	296	41.9	52	17.6	244	82.4	
**Exclusive breastfeeding**^**a**^							< 0.001*
No	118	16.8	89	75.4	29	24.6	
Yes	97	13.7	13	13.4	84	86.6	
Children ≥ 24 months	491	69.5	250	50.9	241	49.1	
**Duration of breastfeeding**							< 0.001*
Currently breastfeeding	120	17.0	29	24.2	91	75.8	
< 23 months	238	33.7	198	83.2	40	16.8	
Up to 23 months	348	49.3	125	35.9	223	64.1	
**Duration of breastfeeding**^**a**^							< 0.001*
Currently breastfeeding	59	8.4	16	27.1	43	72.9	
< 23 months	63	8.9	53	84.1	10	15.9	
Up to 23 months	93	13.2	33	35.5	60	64.5	
Children ≥ 24 months	491	69.5	250	50.9	241	49.1	
**Minimum dietary diversity**							< 0.001*
< 4 food groups	340	48.2	271	79.7	69	20.3	
≥ 4 food groups	366	51.8	81	22.1	285	77.9	
**Minimum dietary diversity**^**a**^							
< 4 food groups	102	14.4	79	77.5	23	22.5	< 0.001*
≥ 4 food groups	113	16.1	23	20.4	90	79.6	
Children ≥ 24 months	491	69.5	250	50.9	241	49.1	
**Feeding frequency**							< 0.001*
Inadequate feeding frequency	378	53.5	283	74.9	95	25.1	
Adequate feeding frequency	328	46.5	69	21.0	259	79.0	
**Feeding frequency**^**a**^							
Inadequate feeding frequency	112	15.9	83	74.1	29	25.9	< 0.001*
Adequate feeding frequency	103	14.6	19	18.4	84	81.6	
Children ≥ 24 months	491	69.5	250	50.9	241	49.1	
**Food restriction during pregnancy**							< 0.001*
Yes	254	36.0	217	85.4	37	14.6	
No	452	64.0	135	29.9	317	70.1	
**Food restriction during pregnancy**^**a**^							
Yes	76	10.8	67	88.2	9	11.8	< 0.001*
No	139	19.7	35	25.2	104	74.8	
Children ≥ 24 months	491	69.5	250	50.9	241	49.1	
**Food restriction during breastfeeding**							< 0.001*
Yes	147	20.8	121	82.3	26	17.7	
No	559	79.2	231	41.3	328	58.7	
**Food restriction during breastfeeding**^**a**^							
Yes	45	6.4	38	84.4	7	15.6	< 0.001*
No	170	24.1	64	37.6	106	62.4	
Children ≥ 24 months	491	69.5	250	50.9	241	49.1	
**Prelacteal feeding**							< 0.001*
Yes	458	64.9	262	57.2	196	42.8	
No	248	35.1	90	36.3	158	63.7	
**Prelacteal feeding**^**a**^							
Yes	154	21.8	89	57.8	65	42.2	< 0.001*
No	61	8.7	13	21.3	48	78.7	
Children ≥ 24 months	491	69.5	250	50.9	241	49.1	
**Food restriction for the child**							< 0.001*
Yes	47	6.7	45	95.7	2	4.3	
No	659	93.3	307	46.6	352	53.4	
**Food restriction for the child**^**a**^							
Yes	17	2.4	16	94.1	1	5.9	< 0.001*
No	198	28.1	86	43.4	112	56.6	
Children ≥ 24 months	491	69.5	250	50.9	241	49.1	
Vaccination status							
**Basic vaccination status**							< 0.001*
Complete	567	80.3	259	45.7	308	54.3	
Incomplete	139	19.7	93	66.9	46	33.1	
Community factors							
**Place of residence**							< 0.001*
Rural	275	39.0	216	78.5	59	21.5	
Urban	431	61.0	136	31.6	295	68.4	

* = statistically significant at 0.05; ^a^ = results of descriptive statistics and chi-squared tests obtained from 215 children under 24 months

In terms of feeding practices, early initiation of breastfeeding within one hour after birth accounted for 61.3% of the children. Approximately 42% of the children were exclusively breastfed. Almost half of the children (49.3%) had a breastfeeding duration of up to 23 months. Regarding food restriction, 36% of the mothers reported having food restrictions during pregnancy. Approximately 65% of the children received prelacteal feeding. Regarding vaccination status, 80.3% of the children had a complete basic vaccination status. Last, in terms of the community factor, 39% of the children lived in rural areas.

In addition, the chi-squared test results showed that all independent variables were significant for stunting. The overall pattern showed that a low sociodemographic status, inappropriate feeding practices, an incomplete basic vaccination status, and living in rural areas were related to stunting.

Specifically, in terms of sociodemographic factors, the prevalence of stunting was significantly higher among older children aged 24 to 59 months, males, children with LBW, children with a birth interval < 24 months, children with mothers with a low education level, children of working mothers, and children living in a low-income household.

In terms of feeding practices, a higher prevalence of stunting was found among children with delayed breastfeeding initiation, children with nonexclusive breastfeeding, children with a duration of breastfeeding < 23 months, children with a dietary diversity of < 4 food groups, and children with inadequate feeding frequency practices. In addition, regarding traditional feeding practices, children of mothers who had food restrictions during pregnancy, children of mothers with food restrictions while breastfeeding, children with food restrictions, and children who received prelacteal feeding had a higher prevalence of stunting. The same pattern was observed among children aged 6 to 23 months.

Regarding vaccination status and the community factor, a higher prevalence of stunting was found among children with incomplete basic vaccinations and those living in rural areas.

### Binary logistic regression analysis of factors associated with stunting

The results of the BLR analysis are presented in Table 2. The Hosmer‒Lemeshow goodness-of-fit test did not show a lack of fit for the model, with a *p* value of 0.090. In the results, a statistically significant relationship was found with four sociodemographic factors (child age, child size, preceding birth interval, and household income level), four feeding practices (early initiation of breastfeeding, exclusive breastfeeding, duration of breastfeeding, feeding frequency), three traditional feeding practices (food restrictions during pregnancy, food restrictions for the child, and prelacteal feeding), vaccination status, and the community factor. The results of statistically insignificant variables are not presented in Table 2.

**Table 2:** Binary logistic regression analysis of factors associated with stunting among children aged 6–23 months and 6–59 months

Variables	Children with stunting 6–23 months (*N* = 215)				Children with stunting 6–59 months (*N* = 706)			
	**Unadjusted OR [95% CI]**	***p* value**	**Adjusted OR [95% CI]**	***p* value**	**Unadjusted OR [95% CI]**	***p* value**	**Adjusted OR [95% CI]**	***p* value**
Sociodemographic factors								
**Child age**								
6–11 months^a^	1.00		1.00		1.00		1.00	
12–23 months	1.33 [0.72–2.43]	0.361	9.81 [1.45–66.67]	0.019*	1.29 [0.69–2.41]	0.432	3.02 [0.56–16.42]	0.200
24–35 months					2.65 [1.43–4.91]	0.002*	8.98 [1.40–57.60]	0.021*
36–59 months					2.24 [1.25–4.02]	0.007*	5.44 [0.88–33.83]	0.069
**Child sex**								
Male	1.28 [0.75–2.20]	0.362			1.47 [1.10–1.98]	0.010*		
Female^a^	1.00				1.00			
**Child size**								
LBW (< 2.5 kg)	15.28 [4.50–51.93]	< 0.001*	18.62 [1.29–268.74]	0.032*	28.29 [10.25–78.07]	< 0.001*	12.55 [2.90–54.21]	< 0.001*
Normal birth weight (≥ 2.5 kg)^a^	1.00		1.00		1.00		1.00	
**Preceding birth interval**								
< 24 months	10.42 [2.09–51.92]	0.004*			17.04 [5.88–49.36]	< 0.001*	15.83 [3.46–72.56]	< 0.001*
≥ 24 months	1.82 [0.96–3.46]	0.068			1.86 [1.33–2.59]	< 0.001*	1.02 [0.51–2.02]	0.963
No previous birth^a^	1.00				1.00		1.00	
**Maternal education level**								
Low (junior high school or below)	23.64 [9.23–60.52]	< 0.001*			23.16 [13.83–38.80]	< 0.001*		
Middle (high school)	3.80 [1.76–8.20]	< 0.001*			3.56 [2.33–5.45]	< 0.001*		
High (college or above)^a^	1.00				1.00			
**Maternal working status**								
Yes	1.23 [0.72–2.11]	0.442			1.36 [1.01–1.83]	0.042*		
No^a^	1.00				1.00			
**Household income level**								
Low (≤ 2 million IDR)	16.46 [7.36–36.81]	< 0.001*			20.99 [13.18–33.44]	< 0.001*	3.51 [1.36–9.06]	0.010*
Middle (> 2 million– < 3.5 million IDR)	2.83 [1.26–6.35]	0.012*			2.57 [1.64–4.03]	< 0.001*	1.35 [0.60–3.03]	0.464
High (≥ 3.5 million IDR)^a^	1.00				1.00		1.00	
Feeding practices								
**Early initiation of breastfeeding**								
After 1 h	2.94 [1.68–5.13]	< 0.001*			2.25 [1.65–3.07]	< 0.001*	2.65 [1.38–5.10]	0.004*
Within 1 hour^a^	1.00				1.00		1.00	
**Exclusive breastfeeding**								
No	19.83 [9.66–40.70]	< 0.001*	7.42 [1.16–47.46]	0.034*	12.80 [8.83–18.54]	< 0.001*	3.19 [1.57–6.50]	0.001*
Yes^a^	1.00		1.00		1.00		1.00	
**Duration of breastfeeding**								
Currently breastfeeding	0.68 [0.33–1.38]	0.283	0.12 [0.02–0.98]	0.048*	0.50 [0.32–0.79]	0.019*	0.51 [0.16–1.68]	0.271
< 23 months	9.64 [4.34–21.41]	< 0.001*	0.92 [0.13–6.37]	0.930	8.83 [5.90–13.23]	< 0.001*	3.38 [1.63–7.00]	0.001*
Up to 23 months^a^	1.00		1.00		1.00		1.00	
**Minimum dietary diversity**								
< 4 food groups	13.44 [7.00–25.80]	< 0.001*			13.82 [9.63–19.84]	< 0.001*		
≥ 4 food^a^ groups	1.00				1.00			
**Feeding frequency**								
Inadequate feeding frequency	12.65 [6.59–24.32]	< 0.001*	12.06 [1.55–93.52]	0.017*	11.18 [7.86–15.91]	< 0.001*	3.79 [1.90–7.57]	< 0.001*
Adequate feeding frequency^a^	1.00		1.00		1.00		1.00	
**Food restriction during pregnancy**								
Yes	22.12 [10.00–48.95]	< 0.001*	17.55 [2.86–107.80]	0.002*	13.77 [9.21–20.60]	< 0.001*	5.87 [3.03–11.38]	< 0.001*
No^a^	1.00		1.00		1.00		1.00	
**Food restriction during breastfeeding**								
Yes	8.99 [3.79–21.33]	< 0.001*			6.61 [4.19–10.43]	< 0.001*		
No^a^	1.00				1.00			
**Prelacteal feeding**								
Yes	5.06 [2.53–10.09]	< 0.001*	10.58 [2.06–54.41]	0.005*	2.35 [1.71–3.23]	< 0.001*	4.27 [2.16–8.41]	< 0.001*
No^a^	1.00		1.00		1.00		1.00	
**Food restriction for the child**								
Yes	20.84 [2.71–160.21]	0.004*			25.80 [6.21–107.22]	< 0.001*	7.74 [1.22–49.16]	0.030*
No^a^	1.00				1.00		1.00	
Vaccination status								
**Basic vaccination status**								
Incomplete	2.70 [1.39–5.24]	0.003*			2.40 [1.63–3.55]	< 0.001*	4.90 [1.26–19.05]	0.022*
Complete^a^	1.00				1.00		1.00	
Community factor								
**Place of residence**								
Rural	15.43 [7.49–31.80]	< 0.001*	83.26 [10.99–630.38]	< 0.001*	7.94 [5.58–11.30]	< 0.001*	7.59 [3.94–14.63]	< 0.001*
Urban^a^	1.00		1.00		1.00		1.00	
**H–L Goodness-of-Fit test**								
Chi-squared test (DF)	70.14 (8)				13.78 (8)			
*p* value	< 0.001				0.090			

*statistically significant at 0.05, *H–L* Hosmer–Lemeshow, *DF* Degree of freedom, *OR* Odds ratio, *CI* Confidence interval, *a* Reference

Specifically, in terms of sociodemographic factors, the odds ratio indicated that children aged 24–35 months (adjusted odds ratio [AOR] = 8.98, 95% confidence interval [CI]: 1.40, 57.60) were more likely to have stunting than those aged 6–11 months. However, the prevalence of stunting in children aged 12–23 months and those aged 36–59 months was not significantly different from that of those aged 6–11 months.

Children with LBW (AOR = 12.55, 95% CI: 2.90, 54.21) were more likely to have stunting than those with normal birth weights. Children with a birth interval < 24 months (AOR = 15.83, 95% CI: 3.46, 72.56) were more likely to have stunting than their counterparts without previous birth. However, the prevalence of stunting in children with birth intervals ≥ 24 months was not significantly different from that of those without previous birth. Children in low-income households (AOR = 3.51, 95% CI: 1.36, 9.06) were more likely to have stunting than children in high-income households. However, the stunting prevalence in children in middle-income households was not significantly different from that in children in high-income households.

In terms of feeding practices, children for whom breastfeeding initiation was delayed (AOR = 2.65, 95% CI: 1.38, 5.10) were more likely to have stunting than children for whom breastfeeding was initiated within one hour after birth. Children with nonexclusive breastfeeding (AOR = 3.19, 95% CI: 1.57, 6.50) were more likely to develop stunting than children with exclusive breastfeeding. Children with a breastfeeding duration of < 23 months (AOR = 3.38, 95% CI: 1.63, 7.00) were more likely to have stunting than children with a breastfeeding duration of up to 23 months.

Children with inadequate feeding frequency (AOR = 3.79, 95% CI: 1.90, 7.57) were more likely to have stunting than their counterparts with adequate feeding frequency. Regarding traditional feeding practices, mothers with food restrictions during pregnancy (AOR = 5.87, 95% CI: 3.03, 11.38) were more likely to have children with stunting than those without food restriction. Children with prelacteal feeding (AOR = 4.27, 95% CI: 2.16, 8.41) were more likely to have stunting than their counterparts without prelacteal feeding. Children with food restriction (AOR = 7.74, 95% CI: 1.22, 49.16) were more likely to have stunting than those without food restriction.

A similar pattern was revealed among children aged 6 to 23 months. Specifically, children with nonexclusive breastfeeding (AOR = 7.42, 95% CI: 1.16, 47.46) were more likely to develop stunting than children with exclusive breastfeeding. Children who were currently breastfeeding (AOR = 8.33, 95% CI: 1.02, 50.00) were less likely to have stunting than children with a breastfeeding duration of up to 23 months. Children with inadequate feeding frequency (AOR = 12.06, 95% CI: 1.55, 93.52) were more likely to have stunting than their counterparts with adequate feeding frequency. Regarding traditional feeding practices, mothers with food restrictions during pregnancy (AOR = 17.55, 95% CI: 2.86, 107.80) were more likely to have children with stunting than those without food restriction. Children with prelacteal feeding (AOR = 10.58, 95% CI: 2.06, 54.41) were more likely to have stunting than their counterparts without prelacteal feeding. In addition, while early initiation of breastfeeding and food restriction for children were related to stunting among children aged 6 to 59 months, they were not related to stunting among children aged 6 to 23 months.

Regarding vaccination status, children with an incomplete basic vaccination status (AOR = 4.90, 95% CI: 1.26, 19.05) were more likely to have stunting than children with a complete basic vaccination status. Last, in terms of the community factor, children living in rural areas (AOR = 7.59, 95% CI: 3.94, 14.63) were more likely to have stunting than their counterparts living in urban areas.

### Reasons for traditional feeding practices

Table 3 presents the results for the type of food and the reasons for food restrictions during pregnancy. Due to multiple answer questions, the mothers could choose more than one type of food and reason. For example, mothers who chose milk could also choose seafood and red meat for food restrictions during pregnancy. Likewise, multiple reasons for food restriction could be selected.

**Table 3:** The type of food and the reasons for food restrictions during pregnancy

Type of food and reasons	Overall *N* = 254	Children with stunting *n* = 217 (85.4%)	Children without stunting *n* = 37 (14.6%)
**Milk**	**224 (88.2%)**	**187 (86.2%)**	**37 (100.0%)**
The baby will be born dirty and smell bad if the mother drinks milk during pregnancy	147 (65.6%)	114 (61.0%)	33 (89.2%)
Drinking milk during pregnancy can cause stomach disorders and nausea for the mother	224 (100.0%)	187 (100.0%)	37 (100.0%)
Red meat (beef, mutton)	**24 (9.4%)**	**24 (11.1%)**	-
Eating meat during pregnancy can cause a miscarriage	13 (54.2%)	13 (54.2%)	-
Eating meat during pregnancy can cause hypertension	16 (66.7%)	16 (66.7%)	-
Eating meat during pregnancy can cause bleeding during delivery	13 (54.2%)	13 (54.2%)	-
Eating meat during pregnancy will change the taste and smell of breast milk	3 (12.5%)	3 (12.5%)	-
Seafood (fish, shrimp, squid)	**22 (8.7%)**	**22 (10.1%)**	-
Eating squid during pregnancy will cause complications during delivery	6 (27.3%)	6 (27.3%)	-
Eating seafood during pregnancy will change the taste and smell of breast milk	2 (9.1%)	2 (9.1%)	-
Eating fish during pregnancy can cause miscarriage	2 (9.1%)	2 (9.1%)	-
Other, maternal allergy to seafood	17 (77.3%)	17 (77.3%)	-
Chicken	**6 (2.4%)**	**6 (2.8%)**	-
Eating chicken during pregnancy will change the taste and smell of breast milk	2 (33.3%)	2 (33.3%)	-
Other, the mother does not like eating chicken	4 (66.7%)	4 (66.7%)	-
Eggs	**5 (2.0%)**	**5 (2.3%)**	-
Eating eggs during pregnancy can cause the baby to develop allergies	3 (60.0%)	3 (60.0%)	-
Eating eggs during pregnancy will change the taste and smell of breast milk	1 (20.0%)	1 (20.0%)	-
Other, maternal allergy to eggs	1 (20.0%)	1 (20.0%)	-

The results showed that of the total study sample, 254 mothers (36%) reported food restrictions during pregnancy. Food restriction was higher among mothers with children with stunting (*n* = 217, 85.4%) than among mothers with children without stunting (*n* = 37, 14.6%). Among the 254 mothers, milk (*n* = 224, 88.2%), red meat (*n* = 24, 9.4%) and seafood (*n* = 22, 8.7%) were the most common food restrictions during pregnancy.

Regarding the reasons for milk restriction, all 224 mothers believed that drinking milk during pregnancy could cause stomach disorders and nausea. In addition, 147 (65.6%) mothers who restricted milk during pregnancy also believed that drinking milk could cause the baby to be born dirty and smell bad.

In addition, mothers avoided beef and mutton during pregnancy because the red color of the meat was considered identical to the color of the blood that caused high blood pressure (hypertension) and pregnancy-related problems. Specifically, 16 (66.7%) mothers considered that eating red meat during pregnancy could cause hypertension. More than half of the mothers (54.2%) who restricted red meat also believed that it could cause miscarriage and bleeding complications during delivery.

Allergy was the most common reason among 17 (77.3%) mothers for avoiding seafood. In addition, 6 (27.3%) mothers did not eat squid during pregnancy because they believed that the tentacles of the squid could trap the baby and cause complications during delivery. Approximately 9% of mothers also believed that eating seafood during pregnancy would change the taste and smell of breast milk during the lactation period.

Table 4 presents the results for the type of food and the reasons for food restrictions for children. Of the total study sample, 47 mothers (6.7%) reported food restrictions for children. The number of food restrictions was higher among children with stunting (*n* = 45, 95.7%) than among children without stunting (*n* = 2, 4.3%). For their children, the mothers restricted eggs (*n* = 30, 63.8%) the most, followed by seafood (*n* = 20, 42.6%).

**Table 4:** Type of food and the reasons for food restrictions for children

Type of food and reasons	Overall *N* = 47	Children with stunting *n* = 45 (95.7%)	Children without stunting *n* = 2 (4.3%)
Eggs	**30 (63.8%)**	**29 (64.4%)**	**2 (100.0%)**
Eating eggs can cause the child to develop an allergy	30 (100.0%)	29 (100.0%)	2 (100.0%)
Seafood (fish, shrimp, squid)	**20 (42.6%)**	**19 (42.2%)**	**1 (50.0%)**
Eating shrimp can cause the child to develop an allergy	13 (65.0%)	12 (63.2%)	1 (100.0%)
The child did not like to eat fish	20 (100.0%)	19 (100.0%)	1 (100.0%)

Mothers avoided giving eggs to their children, as they believed it could cause allergies. Regarding the reasons for restricting seafood, all 20 mothers mentioned that their children did not like to eat fish, and 13 (65.0%) mothers believed that shrimp could cause allergies.

Table 5 presents the type of prelacteal food and the reasons for prelacteal feeding. The results showed that of the total study sample, 458 mothers (64.9%) gave prelacteal foods to their newborns in the first three days of life. Prelacteal feeding was higher among children with stunting (*n* = 262, 57.2%) than among children without stunting (*n* = 196, 42.8%). The prelacteal foods provided by mothers to their children were honey (*n* = 211, 46.1%), coffee (*n* = 155, 33.8%), sugar (*n* = 149, 32.5%), drinking water (*n* = 64, 14.0%), and rice water (*n* = 29, 6.3%).

**Table 5:** Type of prelacteal food and reasons for prelacteal feeding

Type of prelacteal food and reasons	Overall *N* = 458	Children with stunting *n* = 262 (57.2%)	Children without stunting *n* = 196 (42.8%)
Honey	**211 (46.1%)**	**119 (45.4%)**	**92 (46.9%)**
Honey is a reflection of a child’s good life in the future	211 (100.0%)	119 (100.0%)	92 (100.0%)
Keeps the baby’s lips moist and turns the baby’s lips pink naturally	101 (47.9%)	70 (58.8%)	31 (33.7%)
To boost the child’s immune system	205 (97.2%)	119 (100.0%)	86 (93.5%)
Coffee	**155 (33.8%)**	**114 (43.5%)**	**41 (20.9%)**
To prevent seizures or convulsions in the child	155 (100.0%)	114 (100.0%)	41 (100.0%)
Sugar	**149 (32.5%)**	**103 (39.3%)**	**46 (23.5%)**
Sugar is a reflection of a child’s good life in the future	149 (100.0%)	103 (100.0%)	46 (100.0%)
Drinking water	**64 (14.0%)**	**47 (17.9%)**	**17 (8.7%)**
To clean the infant’s intestines, throat, and mouth	64 (100.0%)	47 (100.0%)	17 (100.0%)
Keep the baby’s lips moist	37 (57.8%)	25 (53.2%)	12 (70.6%)
Rice water	**29 (6.3%)**	**24 (9.2%)**	**5 (2.6%)**
To make infants full because the white color and thick texture is similar to that of breast milk	29 (100.0%)	24 (100.0%)	5 (100.0%)
For the child’s growth	29 (100.0%)	24 (100.0%)	5 (100.0%)

Regarding the reasons for providing honey, all mothers believed that applying something sweet, such as honey, would bring good fortune to the child. Approximately 97% of these mothers also mentioned that giving honey to their newborn would boost their immune system, and 47.9% mentioned that applying honey kept their baby’s lips moist and naturally turned their lips pink. Likewise, for honey, all mothers who applied sugar to their baby’s lips believed that sugar would bring the child good fortune.

In addition, mothers who gave coffee to their newborns believed it could prevent convulsions in the child. In terms of reasons for feeding drinking water to their newborns, mothers believed that it could clean the newborn’s intestine, throat, and mouth after breastfeeding, and 37 (57.8%) of these mothers also mentioned that giving drinking water kept the baby’s lips moist. Interestingly, 29 (6.3%) mothers gave rice water to their newborns because it has a texture similar to that of breast milk and to help the child grow.

### Reasons for incomplete vaccination

Table 6 presents the reasons for incomplete basic vaccination among children. The results showed that 139 children (19.7%) in the total study sample had incomplete basic vaccinations. Incomplete basic vaccination was higher among children with stunting (*n* = 93, 66.9%) than among children without stunting (*n* = 46, 33.1%).

**Table 6:** Reasons for incomplete vaccination

Reasons for incomplete vaccination	Overall *N* = 139	Children with stunting *n* = 93 (66.9%)	Children without stunting *n* = 46 (33.1%)
The mother did not want their child to become sick after vaccination	120 (86.3%)	78 (83.9%)	42 (91.3%)
The child did not need to receive vaccines because their body can produce their own immunity	115 (82.7%)	78 (83.9%)	37 (80.4%)
The mother missed the scheduled PHCU vaccination	23 (16.6%)	19 (20.4%)	4 (8.7%)
Vaccines contain ingredients that are not allowed according to religion	14 (10.1%)	11 (11.8%)	3 (6.5%)
Religious teachings did not support vaccination	10 (7.2%)	7 (7.5%)	3 (6.5%)
Other, vaccine was unavailable	9 (6.5%)	7 (7.5%)	2 (4.4%)

Most mothers (86.3%) reported that their child would have fever as a side effect of vaccination, and they did not want the child to become sick. In addition, 82.7% of the mothers believed that their child did not need to receive vaccines because their body can produce natural immunity. Approximately 17% of the mothers in the study sample missed the scheduled PHCU vaccinations, resulting in incomplete vaccination.

As expected, certain religious beliefs, including that vaccines contain porcine-derived ingredients that are not allowed according to religion (10.1%) and religious teachings not supporting vaccination (7.2%), were the other reasons given for incomplete vaccination. In addition, 6.5% of incomplete vaccinations were due to the unavailability of the vaccine, as the COVID-19 pandemic affected the availability and distribution of basic vaccines for children under 9 months of age. Furthermore, the government focused more on providing the COVID-19 vaccine.

## Discussion

The results showed that low sociodemographic status (child age, child size, preceding birth interval, and household income level), inappropriate feeding practices (early initiation of breastfeeding, exclusive breastfeeding, duration of breastfeeding, and feeding frequency), traditional feeding practices (food restrictions during pregnancy, food restrictions for the child, and prelacteal feeding), incomplete basic vaccination, and residence in rural areas were related to stunting.

Traditional feeding practices and incomplete basic vaccination were based on cultural and religious beliefs. The study findings revealed that these practices were higher among children with stunting than among children without stunting. For example, mothers believed that red meat could cause high blood pressure; thus, it could trigger miscarriage or bleeding during delivery. A similar pattern was observed for the vaccination status. Most mothers believed that vaccines were harmful to their children due to their general side effects, such as fever. Another reason was hesitancy in vaccines among mothers due to its ingredients which are not permitted by their religion.

Based on the findings, we emphasize the importance of education for mothers to correct misconceptions of traditional behaviors. The government has recommended that all PHCUs offer counseling to educate mothers about appropriate feeding practices and vaccination. However, PHCUs have to cover all community members within subdistricts, and there are a large number of patients visiting every day; hence, the waiting time is long due to the shortage of health care professionals [36, 37]. Consequently, counseling sessions to educate mothers are often short, and services are limited to a basic physical examination. Therefore, the government should consider gradually improving resources as a long-term strategy to prevent stunting.

As a short-term strategy, the government can utilize local health cadres to monitor traditional feeding practices among mothers and provide education to correct misconceptions. In addition, public relations should continue to improve knowledge and attitudes on appropriate feeding practices and vaccination through mass media or internet-based social media networks. This practice should also be targeted at other family members, particularly grandmothers, as senior family members significantly influence the traditional behavior of mothers in Indonesia [38, 39].

This study focused on inappropriate traditional feeding practices related to stunting. The government should also explore traditional feeding practices that may have benefits in reducing stunting. For example, most pregnant mothers believed that eating *Moringa oleifera* leaves, which are known to contain minerals and micronutrients beneficial for the health of mothers, could prevent LBW [40]. Therefore, through national investigation, a list of inappropriate and appropriate feeding practices can be developed to provide a better understanding of stunting prevention in Indonesia.

In addition, in terms of sociodemographic factors, this study found that children aged 6–11 months were less likely to have stunting than those aged 36–59 months. This could be because the children did not receive adequate feeding to meet their nutritional needs as they aged [23]. Furthermore, studies mentioned that increasing child age was also associated with susceptibility to recurrent infections, which led to an increased risk of stunting [41–43]. The risk of stunting with increased age was reported in previous studies conducted in Tanzania, Madagascar, and Nepal [13, 44, 45].

Our study showed that children with LBW had a higher risk of stunting. Similar studies found that the birth weight of children was significantly associated with stunting [18, 46, 47]. This can be explained by the fact that children with LBW are born with low vital growth nutrients and therefore cannot reach a normal size during childhood [20, 48]. In addition, a previous study in Indonesia revealed children with LBW are more vulnerable to infections, such as diarrhea and acute respiratory infection, which puts them at a higher risk of stunting [12]. This finding reflects the importance of enhancing mothers' knowledge of maternal nutrition during pregnancy to prevent children from having LBW.

We found that children born with a birth interval < 24 months were at high risk of stunting. This could be because short birth intervals negatively affect children’s nutritional needs due to the inability of mothers to provide optimal feeding (e.g., shorter duration of breastfeeding), competition for available food resources, and limited time for mothers to care for each child [49, 50]. This finding was consistent with previous studies conducted in Ethiopia, Congo, and Indonesia [16, 17, 51]. Therefore, family planning interventions are important to prevent stunting.

The study showed that children in low-income households had a higher probability of stunting. This may be attributed to the fact that poor households are likely to have less financial ability to purchase nutritious foods, insufficient food intake, and poor sanitation, which leads to a higher risk of infection, as well as a lack of access to and utilization of health care services [52–54]. In addition, mothers in poor households tend to have a lower level of education, which has been associated with a lack of knowledge on child nutrition [47, 55]. Similarly, the association between household income inequalities and stunting has been observed in numerous studies [13, 56, 57]. This finding highlights the importance of poverty reduction interventions for the long-term prevention of stunting by improving food security and access to basic health services for poor households. Special attention should be given to the education of mothers and the improvement of household income levels.

In addition, in terms of feeding practices, this study found that children for whom breastfeeding was initiated within one hour after birth were less likely to have stunting than those for whom breastfeeding initiation was delayed. This is attributed to early breastfeeding initiation practices to ensure that newborns receive colostrum or ‘first milk’, which is considered the first natural immunization for newborns due to it being rich in vitamins and antibodies [16, 19]. In addition, studies have shown that early initiation of breastfeeding positively impacts milk production and the duration of exclusive breastfeeding [19, 58, 59]. Several studies have also reported that early breastfeeding initiation protects children against stunting [17, 19, 60].

Exclusive breastfeeding has been identified as one of the determinants of stunting in children [15]. This study found that children with nonexclusive breastfeeding were more likely to have stunting than their counterparts with exclusive breastfeeding. This could be because exclusive breastfeeding provides all essential nutrients needed for children's growth and immunity during the first six months of life, thus protecting them from stunting. In addition, studies in Bangladesh reported that exclusive breastfeeding eliminates the risk of contamination from the unhygienic preparation of baby formula milk [61, 62]. Similar findings were reported in cross-sectional studies conducted in Ethiopia [20] and Uganda [34], which revealed a higher prevalence of stunting among children with nonexclusive breastfeeding.

In this study, the duration of breastfeeding was found to be significantly associated with stunting. Children with a breastfeeding duration of < 23 months were more likely to have stunting than children with a breastfeeding duration of up to 23 months. This could be because continuing breastfeeding in the second year of life contributes significantly to the intake of key nutrients that are lacking in low-quality complementary foods. This is consistent with studies conducted in Ethiopia [15, 16]. These findings regarding feeding practices demonstrate that there is a need for a continued effort to educate mothers on the importance and benefits of optimal breastfeeding (i.e., early breastfeeding initiation within one hour after birth, exclusive breastfeeding, and continued breastfeeding up to 23 months).

Our study found that the probability of stunting was higher among children with inadequate feeding frequency than among children with adequate feeding frequency. Feeding frequency is a proxy of energy intake [63]. Therefore, adequate feeding frequency is required to meet the necessary level of energy and nutritional needs and prevent deficiencies that could lead to stunting. This finding was consistent with other studies in Ecuador, Tanzania, and Indonesia, which showed that inadequate feeding frequency was significantly associated with stunting in children [64–66].

In terms of vaccination, this study found that children with complete basic vaccination were less likely to have stunting than children with incomplete basic vaccination. This is probably because incomplete vaccination increases the risk of preventable infectious diseases (e.g., pneumonia, measles, diarrhea), which affect children's general health, including their nutritional status. Specifically, a study in South Ethiopia found that recurrent diarrhea reduced appetite and nutrition absorption, resulting in loss of nutrition for children [67]. Thus, a lack of nutrients could expose children to stunting. This finding was consistent with a previous study conducted in Indonesia, Ethiopia, and Madagascar [12, 19, 45].

Regarding the community factor, our study revealed that children living in rural areas were more likely to have stunting than their counterparts living in urban areas. Other studies have similarly found that children living in rural areas were at higher risk of stunting [23, 50]. In the study area, mothers in rural areas mainly work as farmers. Studies found that mothers working as farmers had less time to care for and properly feed their children than nonfarming mothers such as housewives [68, 69]. Therefore, suboptimal breastfeeding and inappropriate complementary feeding were higher among mothers who worked as farmers [15]. In addition, studies have reported that rural areas are likely to have low household income levels [22, 45], poor sanitation [70], and culture-based lifestyles [54, 68]. Therefore, further research is required for stunting prevention interventions in rural areas.

This study has some limitations that need to be addressed. First, the study area only covered Karanganyar District, which is located in Central Java Province and is dominated by Javanese ethnicity. In Indonesia, there are more than one thousand ethnic groups with different cultures and traditional practices. Therefore, larger-scale studies are required to verify whether the findings of this study on traditional behavior can be generalizable to the entire nation of Indonesia. Second, due to the time gap between collecting medical records from the PHCUs (April 2021) and conducting interviews with the mothers (May 2021), there may have been some changes in the knowledge and practices related to feeding practices among the mothers. Although we cross-checked the data with the maternal and child health handbook, particularly for exclusive breastfeeding and vaccination status, the potential changes should be taken into account in future studies.

Third, this study did not standardize anthropometric measures across the PHCUs. Although all PHCUs used the same anthropometric equipment along with the WHO child growth standard guideline manual, the lack of standardization may cause some undetected errors in the results of this study, which must be considered by future studies. Fourth, the difference between the COR and AOR results indicated confounding effects among the independent variables. Future studies should consider potential interactions between independent variables to obtain more precise socioeconomic patterns of stunting. Fifth, the use of a cross-sectional design in this study without balancing sociodemographic characteristics between the children with stunting and children without stunting groups may not precisely capture the association between traditional feeding practices and stunting. The main analysis of this study was performed adjusting for the sociodemographic factors. This adjustment could minimize the bias associated with less comparable characteristics between children with stunting and children without stunting. However, the reliability of the study findings should be further assessed by future studies using a case–control design with a sociodemographically matched sample between the two groups.

Last, this study did not consider calculating the sample size for data collection because the sample population for this study was all children with stunting aged 6 to 59 months in nine subdistricts and an equivalent number of children without stunting in the same subdistricts. That is, the sample size for this study depended on the number of children with stunting aged 6 to 59 months in nine subdistricts. Nevertheless, the sufficiency of this study’s sample size was assessed using the following formula: *n* = (1.96^2^ × p × q) ÷ d^2^ [71]. In this formula, n is the minimal sample size, p is the prevalence of stunting in the study area (28%), q is 1–p, and d is the tolerated error margin of 5%. The formula suggested a minimum of 310 children, which was far less than the total sample size of this study (706 children). However, having a larger sample size than the suggested sample size may over or underestimate the results. Accordingly, the results must be interpreted with caution.

## Conclusions

The present study highlighted that inappropriate traditional feeding practices, including food restrictions during pregnancy, food restrictions for children, and prelacteal feeding, were associated with stunting. In addition, there are multiple determinants of stunting. Children aged 36 to 59 months, children with LBW, children with a short birth interval of < 24 months, children living in low-income households, children for whom breastfeeding initiation was delayed, children with nonexclusive breastfeeding, children with a duration of breastfeeding < 23 months, children with an incomplete basic vaccination status, and children living in rural areas had a higher prevalence of stunting. The findings reflect the importance of education for mothers to correct misconceptions of traditional feeding practices. Local health cadres can be utilized to correct misconceptions of traditional feeding practices. Moreover, gradually increasing the number of health care professionals at PHCUs in every subdistrict can be considered to strengthen counseling services to improve knowledge and attitudes on the appropriate feeding practices of mothers. Additionally, public relations practices through the mass media should continue for family members, especially senior family members, as they influence mothers’ autonomy in decision-making regarding feeding practices in Indonesia.

## Data Availability

The datasets used and/or analyzed during the current study are available from the corresponding author on reasonable request.

## Abbreviations

AOR: Adjusted odds ratio
BCG: Bacillus Calmette-Guerin
BLR: Binary logistic regression
CI: Confidence interval
DHO: District health office
DTP: Diphteria-tetanus-pertusis
HB: Hepatitis B
Hib: Haemophilus Influenzae Type B
IPV: Inactivated Poliovirus Vaccine
Lao PDR: Lao People’s Democratic Republic
LBW: Low birth weight
MR: Measles-rubella
OR: Odds ratio
PHCUs: Primary health care units
UNICEF: United Nations International Children's Emergency Fund
WHO: World Health Organization
